# One Health Approach: Invasive California Kingsnake (*Lampropeltis californiae*) as an Important Source of Antimicrobial Drug-Resistant *Salmonella* Clones on Gran Canaria Island

**DOI:** 10.3390/ani13111790

**Published:** 2023-05-28

**Authors:** Kevin M. Santana-Hernández, Eligia Rodríguez-Ponce, Inmaculada Rosario Medina, Begoña Acosta-Hernández, Simon L. Priestnall, Santiago Vega, Clara Marin, Marta Cerdà-Cuéllar, Ana Marco-Fuertes, Teresa Ayats, Teresa García Beltrán, Pablo A. Lupiola-Gómez

**Affiliations:** 1 Departamento de Patología Animal, Facultad de Veterinaria, Universidad de Las Palmas de Gran Canaria, 35413 Arucas, Spain; kevin.santana106@alu.ulpgc.es (K.M.S.-H.); eligia.rodriguezponce@ulpgc.es (E.R.-P.); inmaculada.rosario@ulpgc.es (I.R.M.); bego.acosta@ulpgc.es (B.A.-H.); 2 Instituto Universitario de Sanidad Animal (IUSA), Facultad de Veterinaria, Universidad de Las Palmas de Gran Canaria, 35413 Arucas, Spain; 3 Department of Pathobiology and Population Sciences, The Royal Veterinary College, Hatfield AL9 7TA, UK; spriestnall@rvc.ac.uk; 4 Facultad de Veterinaria, Instituto de Ciencias Biomédicas, Universidad Cardenal Herrera-CEU, CEU Universities, 46115 Alfara del Patriarca, Spain; clara.marin@uchceu.es (C.M.);; 5 Unitat Mixta d’Investigació IRTA-UAB en Sanitat Animal, Centre de Recerca en Sanitat Animal (CReSA), Campus de la Universitat Autònoma de Barcelona (UAB), 08193 Barcelona, Spain; 6 IRTA, Programa de Sanitat Animal, Centre de Recerca en Sanitat Animal (CReSA), Campus de la Universitat Autònoma de Barcelona (UAB), 08193 Barcelona, Spain; 7 Departamento de Ciencias Clínicas, Facultad de Veterinaria, Universidad de Las Palmas de Gran Canaria, 35413 Arucas, Spain

**Keywords:** *Salmonella*, multi-drug resistance, PFGE, *Lampropeltis californiae*

## Abstract

**Simple Summary:**

The aim of this study was to investigate the invasive species *Lampropeltis californiae* (California kingsnake) as a reservoir of *Salmonella* and its ability to spread different clones of the bacterium with zoonotic potential into the environment, as well as study its antimicrobial resistance patterns in Gran Canaria (Spain). The main results showed that a high diversity of *Salmonella* subsp. *salamae* strains circulate in Gran Canaria with a high prevalence of resistance shown for antimicrobials of public health importance, as summarised in the European Decision 2013/652/EU.

**Abstract:**

The increase in the reptile population has led to a rise in the number of zoonotic infections due to close contact with reptiles, with reptile-associated salmonellosis being particularly relevant. California kingsnake invasion not only threatens the endemic reptile population of the island of Gran Canaria (Spain) but also poses serious public health problems by spreading zoonotic pathogens and their antimicrobial resistance (AMR) to the environment. Thus, the aim of this study was to assess the occurrence, genetic diversity, and AMR among *Salmonella* spp. strains isolated from California kingsnakes in Gran Canaria Island (Spain). Of 73 invasive individuals captured, 20.5% carried *Salmonella* spp., belonging to different subspecies and serovars, with subsp. *salamae* as the most abundant. Pulsed-field electrophoresis showed high genetic diversity among subsp. *salamae* isolates, and among these, 73.3% showed resistance to at least one of the antimicrobials tested. In conclusion, the present study revealed the importance of wild invasive California kingsnakes as reservoirs of drug-resistant *Salmonella* spp. that could pose a direct threat to livestock and humans. Identification of drug-resistant *Salmonella* strains in wildlife provides valuable information on potential routes of transmission that involve risks to public and animal health.

## 1. Introduction

The Canary Islands are considered a hotspot of Atlantic biodiversity due to their strategic geographical location, volcanic origin, and close contact with Africa and Europe [1]. This archipelago has a great diversity of endemic reptiles that form a large part of its terrestrial fauna. However, the endemic reptile species of the Canary Islands are threatened by a growing number of invasive species [2,3].

In recent years, the population of exotic and native reptiles as pets has increased considerably, with a total population of more than 11 million in European households [4]. The population of free-living exotic reptiles has also increased due to their accidental release or escape into the wild [5], becoming a major threat with a huge ecological impact and favouring the spread of pathogens in the environment [6]. Different studies have described how reptiles act as natural reservoirs of *Salmonella* spp. with a prevalence of up to 90% and are able to carry a wide variety of serovars asymptomatically [7,8,9,10]. The increase in the reptile population has led to a rise in the number of zoonotic infections due to close contact with reptiles, with reptile-associated salmonellosis (RAS) being particularly relevant in at-risk populations such as children, the elderly or immunocompromised adults [11,12,13]. In addition, some authors have described the ability of *Salmonella* strains isolated from domestic reptiles to develop antimicrobial resistance (AMR) [14], thus posing a serious threat to free-living reptiles because of their role in the dissemination of AMR in the environment [15]. *Salmonella* is a very complex gender with a most complex taxonomy than other bacteria [16]. *S*. enterica has long been subdivided by differential antibody reactions into serovars [17]. The use of specific antibodies that could identify distinct cell-surface antigens within lipopolysaccharide and flagella has led to the distinction of over 2500 serovars that differ in their antigenic formulas [18]. In addition, *Salmonella* is also subdivided taxonomically into *S*. *enterica*, which contains multiple subspecies, and a separate species, *S*. *bongori* [18].

In this context, the invasion of *Lampropeltis californiae* (California kingsnake) that has occurred in recent years in Gran Canaria (Canary Islands, Spain) is of particular relevance.

The California kingsnake was first detected free-living in Gran Canaria in 1998, and its population has been rising since then, mainly due to the accidental or intentional release of this invasive snake species into the island ecosystem [3,19]. The increase of the California kingsnake has caused a decrease in the population of different reptiles endemic to Gran Canaria because their diet on the island is mainly based on the endemic reptile species: the Gran Canaria gigant lizard (*Gallotia stehlini*), the Gran Canaria skink (*Chalcides sexlineatus*), and Boettger’s wall gecko (*Tarantula boettgeri*) [20]. In fact, in the areas where the California kingsnake lives, the gigant lizard is locally extinct, the skink has reduced its population by almost 83%, and the Boettger´s wall gecko population has been reduced by half [3], causing a serious ecological impact in Gran Canaria [20]. In addition, this California kingsnake invasion also poses serious public health problems by spreading zoonotic pathogens and their AMR to the environment, where they can be transmitted to humans and other animals (wild or domestic), contributing to the growing problem of AMR [21]. 

The World Health Organisation (WHO) has declared AMR and its ability to transmit between different animal species and humans through the environment as one of the ten most significant threats to public health, encompassing this problem under the “One Health” perspective [22], a concept that refers to a global strategy that seeks to increase interdisciplinary collaboration in the health care of people, animals and the environment to develop and implement programs, policies and laws to improve public, animal and environmental health [23].

Pathogens like *Salmonella* spp. can easily acquire resistance genes through contact with commensal bacteria [24], so the high prevalence of RAS combined with this ability of bacteria to acquire AMR leads to salmonellosis caused by AMR- *Salmonella* strains [25]. Therefore, *Salmonella* spp. could entail therapeutic consequences for humans in close contact with reptiles [26].

With this perspective, the aim of this study is to assess the genetic diversity and the AMR patterns among *Salmonella* spp. strains isolated from California kingsnakes in Gran Canaria Island (Spain).

## 2. Materials and Methods

The animals that are part of the study originate from a program for eradicating invasive species by the Government of the Canary Islands (Spanish Royal Degree 216/2019 and Order 336/20). All the procedures used in this study were performed in accordance with Directive 2010/63/EU EEC for animal experiments.

### 2.1. Sample Collection

**Figure 1:**
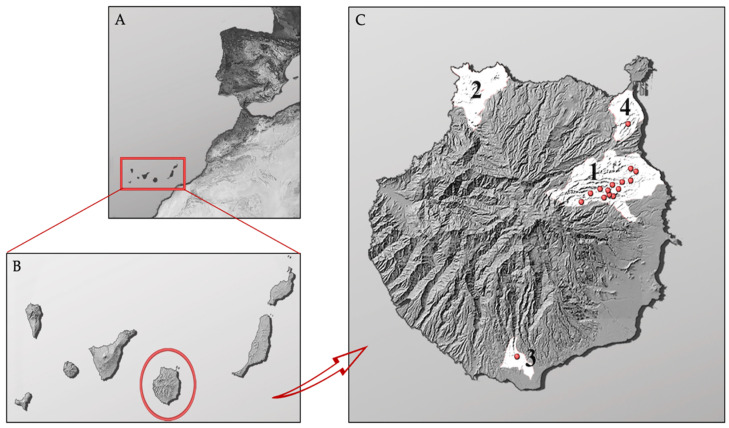
Distribution of California kingsnakes sampled in the island of Gran Canaria. (**A**). Location of the Canary Islands in the Atlantic Ocean; (**B**). Location of the island of Gran Canaria in the Canary Archipelago; (**C**). Representation of the locations where the samples were taken (Nucleus 1, Nucleus 2, Nucleus 3, Nucleus 4). Note: distribution of positive snakes in this study is represented by red dots.

From June to October 2019, a total of 73 individuals of California kingsnake were captured by the staff of *Gestión y Planeamiento territorial y ambiental* (GesPlan) manually and using box-traps in the framework of the eradication project (https://www.gesplan.es/content/orden-33620-que-modifica-la-n%C2%BA-12419-ejecucion-plan-post-life-lampropeltis-y-actuaciones-del, accessed on 1 March 2023). Individuals were captured at four different nuclei in the island of Gran Canaria (1. Main nucleus. 2. Secondary nucleus. 3. Third nucleus. 4. Fourth nucleus, represented in Figure 1). Prior to euthanasia, they were sexed, measured, and weighed. In addition, for *Salmonella* detection, cloacal samples were taken from asymptomatic individuals using sterile cotton swabs immediately after animals were euthanased (Cary–Blair sterile transport swabs, DELTALAB, Barcelona, Spain). The swab was inserted approximately 1 cm into the cloaca to obtain the sample and then kept in Cary–Blair transport medium. All collected samples were transported refrigerated at ≤4 °C to the microbiology laboratory at the Faculty of Veterinary Sciences of the University of Las Palmas de Gran Canaria for microbial analyses within 24 h of collection.

### 2.2. Salmonella Isolation

For *Salmonella* detection, strains were isolated and identified using conventional culture methods as follows: The cloacal swabs were pre-enriched for 24 h in Buffered Peptone water (1:10 vol:vol, Becton, Dickinson and Company, Le Pont de Claix, France (BDC). The pre-enriched samples (100 µL) were transferred onto Rappaport Vassiliadis semisolid agar (MRSV, BDC) at 42 °C for 24 h. The culture obtained onto MRSV was transferred to two different selective agar plates, Xylose Lysine Deoxycholate agar (BDC) and Hektoen Enteric agar (BDC), which were incubated at 37 °C for 24 h. Suspicious colonies that produced sulfhydric acid were selected for biochemical tests (Kliger Iron (BDC), Citrate agar (BDC), Motility–indole (BDC), Phenylalanine agar (BDC), and Voges Proskauer (BDC)). Considering variabilities in the fermentation of lactose by common serovars of subspecies *arizonae* and *diarizonae*, fermenting and non-fermenting colonies were selected. Then, the Analytical Profile Index (API) 20E (BioMérieux, Madrid, Spain) was performed to carry out the confirmation of *Salmonella* spp., following the manufacturer’s indications. Then, to determine the subspecies of *Salmonella* isolates, an analysis was performed following the method proposed by Popoff and Le Minor based on the determination of biochemical characteristics and susceptibility to phage O1 [18]. Finally, confirmed *Salmonella* spp. strains were serotyped in accordance with Kauffman–White–Le Minor technique [18] at the National Reference Laboratory for Animal Health (Algete, Madrid, Spain).

### 2.3. Molecular Typing of Salmonella Isolates

Genotyping of *Salmonella* spp. isolates was performed by pulsed-field gel electrophoresis (PGFE) according to the PulseNet standardised protocol (www.pulsenetinternational.org/protocols/pfge/, accessed on 15 March 2023). The genomic DNA of the isolates was digested with Xbal and BlnI restriction enzymes (Roche Applied Science, Indianapolis, IN). We analysed the resulting PFGE band patterns using Fingerprinting II v3.0 software (Bio-Rad, Hercules, CA, USA). Similarity matrices were calculated using the Dice coefficient with a band position tolerance of 1.5%, and cluster analysis was performed by the unweighted-pair group method with arithmetic mean (UPGMA). A cut-off of 90% was used for the determination of the different profiles (PFGE type or pulsotype).

### 2.4. Antimicrobial Susceptibility Testing

*Salmonella* spp. strains were inoculated onto Müller–Hinton agar (BDC) to form a bacterial lawn; then, antibiotic discs were put on the plates, which were incubated at 37 °C for 24 h. Antimicrobial agents were selected following those set out in Decision 2013/652/EU [27], including two quinolones: ciprofloxacin (CIP; 5 µg) and nalidixic acid (NAL; 30 µg); one aminoglycoside: gentamicin (GEN, 10 μg); one potentiated sulfonamide: trimethoprim-sulfamethoxazole (TRS; 25 µg); one phenicol: chloramphenicol (CHL; 30 µg); one pyrimidine: trimethoprim (TRI; 5 µg); three ß-lactams: ampicillin (AMP; 10 µg), cefotaxime (CTA; 30 µg), ceftazidime (CTZ; 30 µg); one macrolide: azithromycin (AZI; 15 µg); one polymyxin: colistin (COL; 10 µg); and one glycylcycline: tigecycline (TIG; 15 µg). After the 24 h incubation period at 37 °C, the inhibition zone around each disc was measured. These zones were interpreted as susceptible (S) or resistant (R) according to the European Committee on Antimicrobial Susceptibility Testing (EUCAST) indications [28] (http://www.eucast.org/clinical_breakpoints/, accessed on 6 February 2023) for Enterobacteriaceae, and when not possible, Clinical and Laboratory Standards Institute (CLSI) indications were used (https://clsi.org/media/2663/m100ed29_sample.pdf, accessed on 6 February 2023) [29]. Multidrug resistance (MDR) was defined as acquired resistance to at least one agent in three or more antimicrobial classes [30].

### 2.5. Statistical Analysis

A Generalised Linear Model (GLM), which assumed a binomial distribution for *Salmonella* spp. shedding was fitted to the data to determine whether there was an association with the categorical variables (sex, body length, and weight) or not. A *p* ≤ 0.05 was considered to indicate a statistically significant difference. Data are presented as least squares means ± standard error of the least squares means for the body length and weight. In addition, a GLM was performed to assess the serovars isolated in this study. Finally, a GLM was performed to study the relationship between *Salmonella* spp. and their AMR. Analyses were carried out using a commercially available software application (SPSS 24.0 software package; SPSS Inc., Chicago, IL, USA, 2002). 

## 3. Results

During this study, a total of 73 California kingsnakes were captured at four different nuclei in the Gran Canaria Island: Nucleus 1 (n = 57), Nucleus 2 (n = 4), Nucleus 3 (n = 4) and Nucleus 4 (n = 8). From all animals sampled, 50.7% (37/73) were females and 49.3% (36/73) were males. The mean body length and weight of the total animals were 922.5 ± 18.4 cm and 264.7 ± 15.0 g, respectively. 

### 3.1. Salmonella spp. Identification and Serotyping

From all animals sampled, 20.5% (15/73) tested positive for *Salmonella* spp. (Table 1). Due to the difference in the number of samples taken between the different nuclei, it was not possible to establish statistical significance among the different nuclei. In addition, statistically significant differences were observed between the presence of *Salmonella* and the sex of the sampled animals, with the presence of *Salmonella* being more prevalent in females than in males (*p* < 0.05). However, no statistically significant differences were observed between the presence of *Salmonella* and the measure and weight of the positive animals (*p* > 0.05). 

All *Salmonella* spp. isolates were classified as *Salmonella enterica* (n = 15). The subspecies isolated were, in decreasing order, *S. salamae* (66.6%, 10/15), *S. enterica* (20.0%, 3/15), *S. diarizonae* (6.7%, 1/15), and *S. houtenae* (6.7%, 1/15). Seven different serovars of *S. enterica* were identified (Table 2). 

### 3.2. Salmonella Molecular Typing

**Figure 2:**
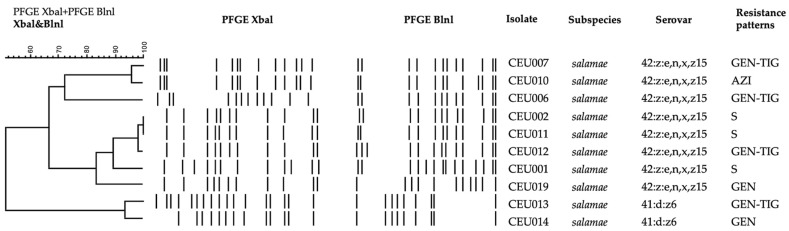
PFGE dendrogram of Xbal and Blnl profiles of *Salmonella enterica* subsp. *salamae*. isolates from California kingsankes in Gran Canaria and their resistance patterns. GEN: gentamicin, TIG: tigecycline, AZI: azithromycin. S: sensible.

PFGE analysis revealed six different pulsotypes within the ten subsp. *salamae* isolates, five of them belonging to serovar 42:z:e,n.x.z15 and the remaining one corresponding to isolates of serovar 41:d:z_6_ (Figure 2). Isolates of ser. 42:z:e,n.x.z15 were grouped in two main clusters at 83.11% and 71.92% similarity, respectively. The single isolates of ser. Cerro and ser. Kentucky were not typable with the enzyme BlnI. 

### 3.3. Antimicrobial Susceptibility

From all strains isolated, 73.3% (11/15) were resistant to at least one of the twelve antimicrobials tested. The highest frequency of AMR was found to GEN (60%, 9/15) and TIG (40%, 6/15), followed by AZI (13.3%, 2/15), and AMP and CTZ with only one strain resistant (6.7%, 1/15) (*p* < 0.001). No resistance was found against CHL, CIP, COL, CTA, NAL, TRS, and TRI (Figure 2). Furthermore, the *S. enterica* ser. Cerro isolate was resistant to three different antimicrobials. 

Overall, four different resistance patterns were found (Table 3, Figure 2). GEN alone (36.4%, 4/11) and GEN-TIG (36.4%, 4/11) were the most frequent patterns observed. Isolates with either of these AMR patterns also showed different PFGE profiles (Figure 2). AZI alone and the combination of GEN-AZI-TIG and AMP-CTZ-TIG were only observed once (9.1%, 1/11). 

## 4. Discussion

The present study demonstrates that 20.5% of invasive California kingsnakes from the island of Gran Canaria (Canary Islands, Spain) carry *Salmonella* spp. Genotyping analysis showed high diversity among isolates of subsp. *salamae*. From these isolates, 73.3% presented resistance to at least one of the antimicrobials tested, included in Decision 2013/652/EU. To our knowledge, this is the first study in the literature assessing the prevalence, genetic relatedness, and AMR of this zoonotic pathogen in the California kingsnake on Gran Canaria Island.

Reptiles have been considered carriers of *Salmonella* spp. worldwide, and their serological variety and antimicrobial resistance have been studied [10,29,31,32]. Therefore, they may pose a danger as a source of dissemination of the bacterium in the environment, as well as an important cause of animal and human infection, especially in at-risk populations [11,29,33]. Different studies worldwide have shown a wide variety of *Salmonella* spp. prevalence in domestic and wild snakes (being less prevalent in the latter), based on the geographic area, reptile species, time of sampling (as shedding is intermittent), and methodology used [31]. This vast difference among studies highlights the poor knowledge about *Salmonella* epidemiology in wild reptiles [31]. *Salmonella* spp. is an enterobacterium that is highly associated with stress. Hence, situations that increase stress in these animals increase the shedding of the bacteria into the environment [11]. Therefore, as has been done in other animals (such as livestock), it seems mandatory to unify methodology in terms of sampling and analysis of samples to be able to compare results between different regions and thus obtain more information from all the research carried out. In line with previous studies, our results showed that California kingsnake carries different subspecies and serovars of *Salmonella* [31] and, as in other studies, our results showed that sex directly influences the shedding of *Salmonella* spp. in reptiles, as the prevalence has been found in our study to be higher in California kingsnake females than in males [34,35]. However, as also seen in other studies, our results showed that size and weight have no influence on *Salmonella* shedding in reptiles [36].

Similarly to other reports, in this study, *Salmonella enterica* was the most common species isolated from reptiles [10,36,37]. The four subspecies we isolated (*S. enterica enterica* [I] (3/15), *S. enterica salamae* [II] (10/15), *S. enterica diarizonae* [IIIb] (1/15) and *S. enterica houtenae* [IV] (1/15)) have also been widely reported in different reptile studies, including snakes, turtles, and lizards [6,10,14,29,32]. In addition, *Salmonella enterica enterica* has been reported in the Gran Canaria Island as the main species isolated from feral cats, although the identified subspecies do not coincide with those found in the California kingsnake [34].

In some studies, snakes have been found to have the greatest diversity of *Salmonella* subspecies [32]. Of all *Salmonella enterica* subspecies, *enterica* has been considered the most prevalent subspecies in reptiles [38,39]. However, we have isolated this subspecies with a low prevalence. Instead, subsp. *salamae* was the most prevalent subspecies isolated in this study (53.3%). One of the hypotheses that could explain this fact is that the Canary Islands are a limited geographical area, which has allowed subsp*. salamae* to colonise these populations. Subsp*. salamae* has previously been isolated from other animals, such as carnivores [40], wild boars [41], and poultry [42]. However, this subspecies has not frequently been associated with human infections [43,44,45]. Finally, subsp*. diarizonae* and subsp*. houtenae* are two species especially related to snakes [46], and these subspecies have also been occasionally found in other animal species [41,47,48]. subsp*. diarizonae* and subsp*. houtenae* have also been described in human cases as a result of the close contact between humans and their exotic pets [49,50,51,52].

*Salmonella* serovars, with major epidemiological importance in human medicine and domestic animals, occur less frequently in cold-blooded animals [31]. To the authors’ knowledge, this is the first report of the isolation of *Salmonella* ser. 42:z:e,n.x.z15 in the world, as well as the first report of *Salmonella* ser. Cerro, Midway, Kentucky, 41:d:z6, 60:-:-, and 43:z4,z23:- from California kingsnakes. Among them, ser. Kentucky has been considered a public health threat [52], as it has been identified as causing human pathologies, such as urinary tract infections or gastroenteritis [53,54]. This serovar has been isolated from a wide range of animals, ranging from pet reptiles (snakes, turtles, or lizards) [55] to poultry, dairy, cattle, or food [56], being an important source of human infections, directly through pets [55] or indirectly through food consumption [56]. Regarding *S.* ser. Cerro, it is a serovar infrequent in humans and widely adapted and associated with cattle pathologies [57,58], but it has also been described in some snake species [59,60] and other reptiles such as lizards [61] and turtles [11]. In turn, *S.* ser. Midway [32,62] and 43:z4,z23:- [9,52,59,61] have been widely reported in different snake species, as they are serovars closely related to snakes. To the authors’ knowledge, the serovar 41:d:z6 has only been reported once, isolated from a green lizard, and it was designed as *S.* Hennepi [63], but no more references have been found. 

The high genetic diversity of subsp. *salamae* isolates, which were all recovered from snakes from the same nuclei, suggests different sources of infection. Nevertheless, a broader sampling and a greater number of isolates to compare would be needed to confirm this. In addition, further studies, including whole genome sequencing (WGS), could be interesting for improving the information related to the epidemiology of *Salmonella* in reptiles [64].

Studies on AMR in *Salmonella* spp. isolates from wild snakes are scarce [15,65], but some studies suggest similar patterns of drug resistance to strains isolated from captive or pet snakes [29,52,66]. In line with previous studies, resistance against GEN was the most common AMR observed [15,65,67], followed by TIG and AZI [68]. This study has also shown resistance against AMP and CTZ [29,52]. Bacterial resistance to these antimicrobials has been widely documented due to its extensive use in veterinary medicine [65,67,68]. Hence, the close contact of these snakes with humans and livestock could explain the high frequency of GEN-resistant strains in this study and, in addition, to TIG and AZI. These results, along with those of other authors, suggest that the problem of AMR is not limited to its initial niches, potentially livestock, but that wildlife could also play an important role in the spread of these strains in households [69]. 

In our study, no resistance against CIP, NAL, CHL, TRS, TRI, COL, and CTA was found in *Salmonella* isolates, as seen in other studies [32,68]. However, there is a huge difference in AMR patterns of *Salmonella* spp. strains depending on the geographical location [31,32,52], ranging from 14% in Taiwan [70] to 90% in Italy [68]. These results demonstrate that external factors other than the animal species could influence the development of AMR patterns. 

## 5. Conclusions

The present study revealed the importance of wild invasive California kingsnakes as reservoirs of *Salmonella* spp., which could pose a direct threat to livestock and humans. Identification of drug-resistant *Salmonella* subsp. and serovars in wildlife provides valuable information on potential routes of transmission that involve risks to public and animal health. 

## Figures and Tables

**Table 1:** Relationship between positive animals and their location, sex, body length, and weight.

		n	% *Salmonella* spp.	*p*-Value
Location	1	57	86.6	
(nuclei)	2	4	0	
	3	4	6.7	
	4	8	6.7	
Sex	Female	37	73.3 ^a^	*p* = 0.042
	Male	36	26.7 ^b^	
Body Length	≤900 cm	33	40.0	*p* = 0.647
	>900 cm	40	60.0	
Weight	≤250 g	39	46.7	*p* = 0.732
	251–500 g	28	40.0	
	>500 g	6	13.3	

n: total number of animals sampled. ^a, b^: different superscripts in each column mean significant differences with a *p*-value < 0.05. %: percentage of *Salmonella* positive animals.

**Table 2:** *Salmonella enterica* serovars isolated from California kingsnake.

Subspecies	Serovar	n	Nuclei	Prevalence (%)
*salamae*	42:z:e,n.x.z15	8	1	53.3 ^a^
	41:d:z6	2	1	13.2 ^b^
*enterica*	Cerro	1	1	6.7 ^b^
	Kentucky	1	3	6.7 ^b^
	Midway	1	1	6.7 ^b^
*diarizonae*	60:-:-	1	4	6.7 ^b^
*houtenae*	43:z4,z23:-	1	1	6.7 ^b^

n: Number of *Salmonella* strains per serovar. %: Percentage of positive samples. Nuclei: 1. Main nucleus, 3. Third nucleus, 4. Fourth nucleus. ^a,b^ Different superscripts in each column mean significant differences with a *p*-value < 0.05.

**Table 3:** Antimicrobial resistance according to the antibiotic and the *Salmonella* species and serovars isolated.

Species	Serovar	n	CIP	AMP	NAL	GEN	CHL	TRS	TRI	COL	CTA	AZI	CTZ	TIG
*S. enterica* subsp. *salamae*	42:z:e,n.x.z15	8	0	0	0	4	0	0	0	0	0	1	0	3
	41:d:z6	2	0	0	0	2	0	0	0	0	0	0	0	1
*S. enterica* subsp. *enterica*	Cerro	1	0	0	0	1	0	0	0	0	0	1	0	1
	Kentucky	1	0	0	0	1	0	0	0	0	0	0	0	0
	Midway	1	0	0	0	1	0	0	0	0	0	0	0	0
*S. enterica* subsp. *diarizonae*	60:-:-	1	0	1	0	0	0	0	0	0	0	0	1	1
*S. enterica* subsp. *houtenae*	43:z4,z23:-	1	0	0	0	0	0	0	0	0	0	0	0	0

n: number of *Salmonella* strains. CIP: ciprofloxacin, AMP: ampicillin, NAL: nalidixic acid, GEN: gentamicin, CHL: chloramphenicol, TRS: trimethoprim-sulphamethoxazole, TRI: trimethoprim, COL: colistin, CTA: cefotaxime, AZI: azithromycin, CTZ: ceftazidime, TIG: tigecycline.

## Data Availability

Not applicable.
